# Supplementary material for: The Construction and Exploration of a Comprehensive MicroRNA Centered Regulatory Network in Foxtail Millet (Setaria italica L.)
Source: Front Plant Sci. 2022 May 6;13:848474. doi: 10.3389/fpls.2022.848474 (PMC9121102; doi:10.3389/fpls.2022.848474)
Supplement: Supplementary file 7 [file Data_Sheet_7.zip › Sit-miR528_Seita.7G243900.1_3736_TPlot.pdf]

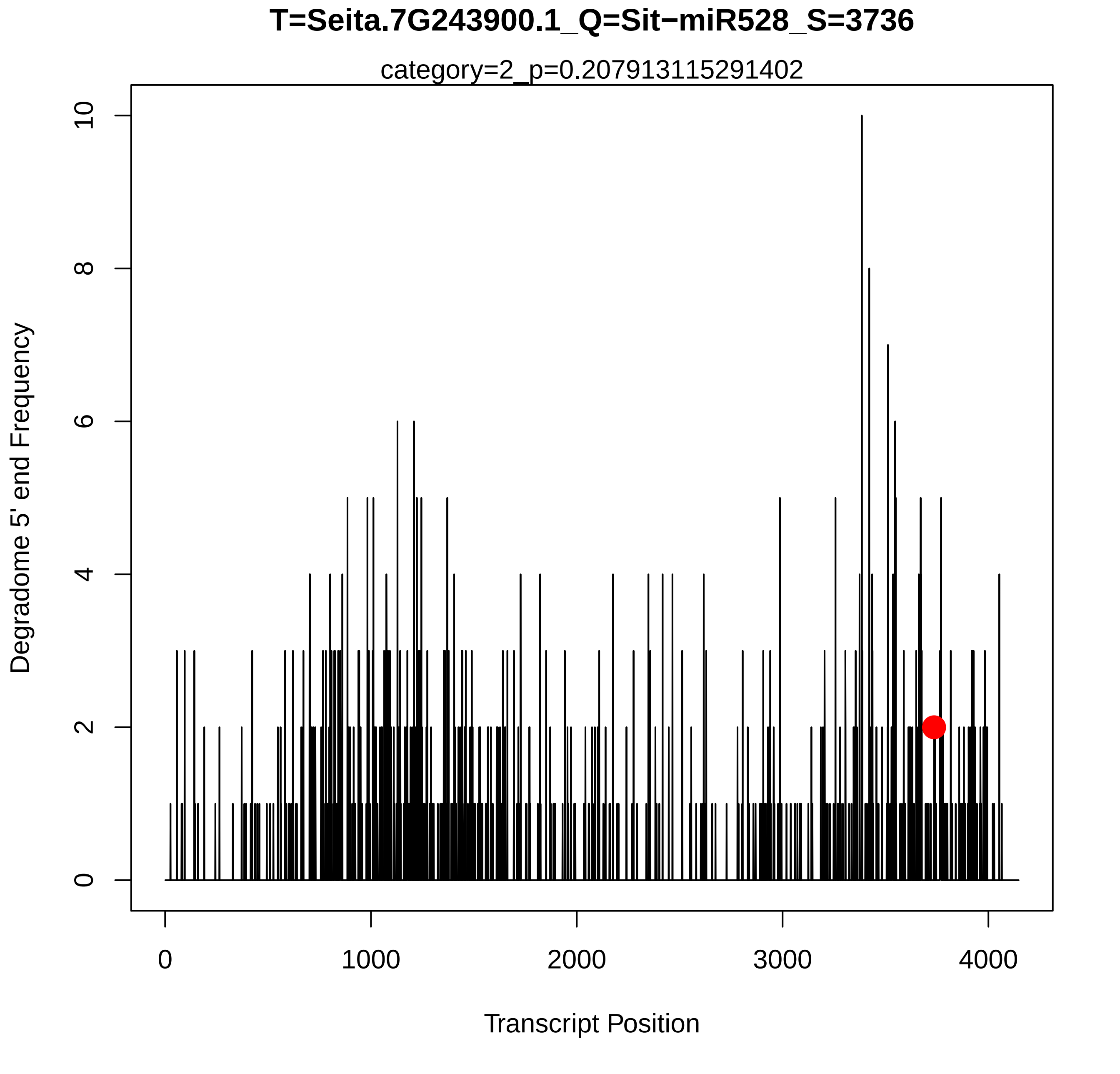
T=Seita.7G243900.1_Q=Sit−miR528_S=3736
category=2_p=0.207913115291402
Degradome 5' end Frequency
Transcript Position
0
2
4
6
8
10
0
1000
2000
3000
4000
